# Nitrogen deposition and multi-dimensional plant diversity at the landscape scale

**DOI:** 10.1098/rsos.150017

**Published:** 2015-04-08

**Authors:** Tobias Roth, Lukas Kohli, Beat Rihm, Valentin Amrhein, Beat Achermann

**Affiliations:** 1 Zoological Institute, University of Basel, Vesalgasse 1, Basel 4051, Switzerland; 2 Hintermann and Weber AG, Austrasse 2a, Reinach 4153, Switzerland; 3 Meteotest, Fabrikstrasse 14, Bern 3012, Switzerland; 4 Air Pollution Control and Chemicals Division, Federal Office for the Environment (FOEN), Bern 3003, Switzerland

**Keywords:** biodiversity monitoring, ecosystem stability, global change, species richness

## Abstract

Estimating effects of nitrogen (N) deposition is essential for understanding human impacts on biodiversity. However, studies relating atmospheric N deposition to plant diversity are usually restricted to small plots of high conservation value. Here, we used data on 381 randomly selected 1 km^2^ plots covering most habitat types of Central Europe and an elevational range of 2900 m. We found that high atmospheric N deposition was associated with low values of six measures of plant diversity. The weakest negative relation to N deposition was found in the traditionally measured total species richness. The strongest relation to N deposition was in phylogenetic diversity, with an estimated loss of 19% due to atmospheric N deposition as compared with a homogeneously distributed historic N deposition without human influence, or of 11% as compared with a spatially varying N deposition for the year 1880, during industrialization in Europe. Because phylogenetic plant diversity is often related to ecosystem functioning, we suggest that atmospheric N deposition threatens functioning of ecosystems at the landscape scale.

## 2. Introduction

Nitrogen (N) is an essential plant nutrient, and many species-rich ecosystems are adapted to conditions of low N availability [1,2]. Increasing N availability in such ecosystems often favours a small number of highly competitive species, resulting in decreasing overall plant diversity through competitive exclusion [3,4]. Atmospheric N deposition leading to an increased N availability is therefore an important element of global change threatening biodiversity [1,5]. Main sources of human atmospheric N emissions are combustion of fuel and of biomass and emissions from fertilizer and manure [1].

So far, evidence for negative effects of N deposition on biodiversity mainly stems from studies conducted on small-scale plots in habitats with low nutrient status and high conservation value [6]. Yet, ecosystems are managed and ecosystem services are delivered at the landscape scale [7,8], and results from small-scale study plots might not easily be scaled up to explain larger scale patterns [9] because responses of plant biodiversity to N enrichment are scale-dependent [10].

Here, we used data from 381 study plots of 1 km^2^ each, covering most habitat types of Central Europe, to examine how six different aspects of plant diversity are related to N deposition. Because geophysical and human influences vary with elevation [11], we used study plots spanning an elevational range of 2900 m, and we also accounted for factors such as agricultural and woodland area. We estimated the loss of plant diversity due to N deposition by comparing model predictions on current and historic species composition under two historic N deposition scenarios. In our first historic scenario, we assumed homogeneously distributed low levels of N deposition, because analyses of herbarium specimens revealed that in pre-industrial times N concentrations were much lower and regional differences were less marked than today [12]. For the second historic scenario, we assumed a modest and spatially varying N deposition predicted for 1880, during industrialization in most European countries [13]. Compared to both historic scenarios, our models suggested that current atmospheric N deposition negatively affects all six aspects of plant diversity at the landscape scale.

## 3. Material and methods

### 3.1 Plant data

Fieldwork took place between 2005 and 2009 in Switzerland. About 70% of Switzerland is mountainous, including the Alps (about 60% of Switzerland) and the Jura Mountains (about 10% of Switzerland). Plant data are from the Swiss biodiversity monitoring indicator ‘species richness in landscapes’ (Z7), which aims to monitor vascular plant diversity at the landscape scale [14,15]. Based on the national coordinate system of 41 285 1 km^2^ cells, a sample grid of 428 regularly spaced study plots, each of 1 km^2^ size, was selected using a randomly chosen starting cell. Excluding study plots of 100% water surface, as well as study plots that were too dangerous for fieldwork because of their ruggedness [16], plant data from 381 study plots were used for the current study.

Qualified botanists who received special training to reduce among-observer variation performed the surveys. For each study plot, occurrences of vascular plants were surveyed along a 2.5 km transect that followed existing trails preferably near the diagonal of the study plots [17]. If no trails existed, surveyors marked the transect route in the field and plotted it on a map. Transects were inspected once in spring and again in summer, assuring that data collection spanned a large variation in flowering phenologies [18]. On 19 sample plots with short vegetation period at high elevations, only one inspection per field season was conducted. During each inspection, surveyors recorded all plant species (presence/absence) within 2.5 m to each side of the transects both on the way forth and back, respectively. The overall detection error was relatively small with an average of 6.6% undetected presences per species as inferred in an earlier study using site-occupancy models [16]. Overall, the plant surveys yielded 93 621 observations of 1768 plant species.

We calculated six measures of plant diversity for each study plot: (i) total species richness (Total SR), (ii) number of species typically found on nutrient-poor soils (Oligo SR; oligotrophic species with N-values of one and two [19]), and (iii) number of target species for which Swiss agriculture has particular responsibility of conservation (Agri SR [20]). We examined (iv) community uniqueness of a study plot by calculating the average Simpson dissimilarity index [21,22] of species composition for the study plot paired with each other plot. A value close to one would indicate a plot with a high proportion of unique species (i.e. a high proportion of relatively rare species); a value close to zero would indicate a high proportion of common species [23]. Such a measure for community uniqueness is based on the concept of ‘differentiation diversity’ [24] and can be interpreted as differences in species composition between plots while controlling for differences in species richness [25]. To calculate (v) functional diversity (FD), we selected traits from the LEDA database [26] or Flora Indicativa [19] that are important for competition and persistence: adult longevity, plant height, presence of reserve or storage organs, root depth, seed longevity and seed mass. As a measure for the functional distances between the species present in a community, we used Gower distances that can be used with both continuous and binary data [27]. Our measure of FD was the sum of functional distances between each pair of species at a study plot [27,28]. Finally, we calculated (vi) phylogenetic diversity (PD) as the sum of the branch lengths for the species present in a community [29]. The branch lengths were obtained from the molecular phylogeny of Durka & Michalski [30] that represents 4685 vascular plant species from Central Europe.

### 3.2 Statistical analyses

Our aim was to examine the effect of atmospheric N deposition on six measures of plant diversity. We therefore used linear and quadratic terms of average N deposition per plot as predictors in linear models (LMs) on the six measures. Atmospheric N deposition (kg N ha^−1^yr^−1^) in 2007 was determined at 0.1×0.1 km cells using a combination of modelling and monitoring approaches as described in an earlier study [31]. For each plot of 1 km^2^, we averaged N deposition values from the cells containing parts of the transect lines used for our plant surveys. In each LM, we additionally used the covariates listed in table 1 with linear and quadratic terms. These covariates were identified in an earlier study examining numerous environmental factors and were contained in the best model predicting Swiss vascular plant diversity at the landscape scale [32]. Total SR, Oligo SR and Agri SR were analysed using generalized linear models with Quasi-Poisson distributions. Community uniqueness, FD and PD were analysed using LMs with normal error distribution. In all cases, inference was based on full LMs without applying model selection, because inference based on full models usually leads to the most conservative results [33].

**Table 1.** Predictor variables used in linear mixed models on plant diversity. Covariates were selected based on an earlier study examining numerous environmental factors to explain the Swiss vascular plant diversity at the landscape scale [32].

description	unit	mean±s.d.	min.	max.
atmospheric N deposition	kg ha^−1^ yr^−1^	17.5±8.3	2	44
elevation	m.a.s.l.	1243±715	263	3175
lowland agricultural area	ha	25.5±29.8	0	97
open woody formation area	ha	6.5±8.9	0	65
calcareous substrate	%	82.1±32.6	0	100
range of temperature variation Jul.–Jan.	°C	5.4±3.9	0.2	18.3
standard deviation of creek lengths per plot	m	30.9±18.6	0	76

To standardize parameter estimates and thus to allow comparison of the effects of average N deposition among the six measures of plant diversity, we then estimated effect sizes (*E*_*i*_) for study plot *i* as follows: for each plant diversity measure, we made model predictions using the current plot-specific N deposition (${\mathrm{Pred}}_{i}^{\mathrm{Current}}$) and model predictions using two different scenarios of historic N deposition. For the first historic scenario (${\mathrm{Pred}}_{i}^{\mathrm{Scenario}\;1}$), we assumed a homogeneous N deposition that was set to 1 kg N ha^−1^ yr^−1^, which is the natural background N deposition without human influence as suggested in [6,34]. For the second historic scenario (${\mathrm{Pred}}_{i}^{\mathrm{Scenario}\;2})$, we selected predicted values of N deposition for the year 1880, using time series that were estimated for the period 1880 to 2000 for the Coordination Centre for Effects (wge-cce.org) of the United Nations Economic Commission for Europe, based on 29 cells of 2500 km^2^ from the grid of the European Monitoring and Evaluation Programme (EMEP) covering Switzerland [35]. Historic emissions were calculated using meteorologically standardized atmospheric source–receptor transfer coefficients derived from the EMEP Lagrangian acid deposition model [13]. Our second scenario thus allowed for a human influence that varied among EMEP grid-cells. To estimate historic N deposition at each of our 1 km^2^ study plots in the second scenario, we estimated separate depositions for NHy and NOx for the year 2000 using the same approach as for estimating atmospheric N deposition in 2007 (see above); we then used the reduction factor for N deposition (N deposition in 2000/N deposition in 1880) as estimated for the respective 2500 km^2^ cell to obtain estimates of N deposition for 1880 for each 1 km^2^ study plot.

For both historic scenarios, we calculated effect sizes as follows:

$$E_{i}=100\times \frac{{\text{Pred}}_{i}^{\mathrm{Current}}-{\text{Pred}}_{i}^{\mathrm{Scenario}\;1\;\mathrm{or}\;2}}{{\text{Pred}}_{i}^{\mathrm{Scenario}\;1\;\mathrm{or}\;2}}.$$

Thus, the effect size for plant diversity change was the estimated value of plant diversity at a 1 km^2^ plot from the model using current N deposition minus the estimated value of plant diversity from the model using historic N deposition based on scenario 1 or 2, in % of the value from the model using historic N deposition. An effect size of −20% would thus suggest a loss of plant diversity of 20% as compared with the respective historic N deposition.

We used the software R [36] for statistical analyses. To estimate parameter values and 95% credible intervals, we used a Bayesian approach; for all parameters, we sampled 2000 values from the posterior distributions using the R-function *sim* [37]. From the sampled values, we calculated the mean effect sizes, and the 2.5 and 97.5% quantiles of all simulated values were our estimates for the 95%-credible intervals of effect sizes. We made semi-variograms [38] on all models and did not identify relevant spatial autocorrelations.

## 4. Results

Average (±s.d.) atmospheric N deposition at the 381 plots was 17.5 (±8.3) kg N ha^−1^ yr^−1^, which is about 17 times the assumed natural atmospheric N deposition without human influence under our first historic scenario assuming a homogeneous N deposition based on values given in Bobbink R & Hettelingh [6] and Butterbach-Bahl [34]. Under the second historic scenario allowing for a modest and spatially varying human influence, we estimated that current atmospheric N deposition is about 2.5 times the assumed atmospheric N deposition in 1880.

**Figure 1.**
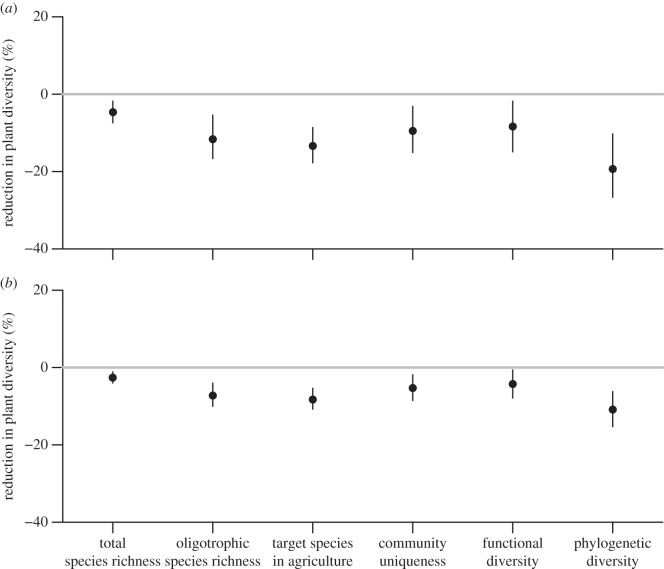
Standardized estimated median effects (and 95% credible intervals) of current atmospheric N deposition on six measures of plant diversity, compared to historic atmospheric N deposition based on (*a*) scenario 1 assuming a homogeneous natural N deposition of 1 kg N ha^−1^ yr^−1^ without human influence and (*b*) scenario 2 assuming a spatially varying N deposition for the year 1880, during industrialization in Europe.

**Figure 2.**
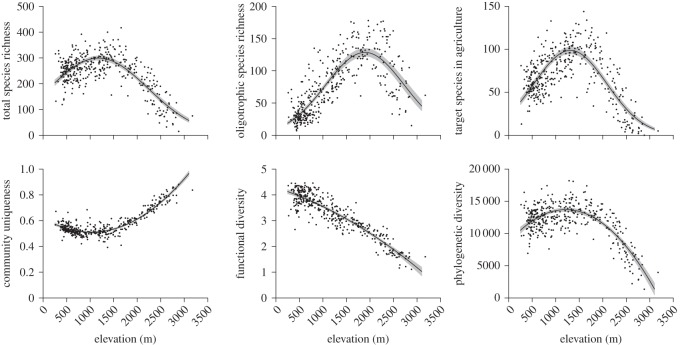
Values of six measures of plant diversity along the elevational range covered in the Swiss Biodiversity Monitoring programme. Points are 381 study plots, black lines are regression lines from LMs with linear, quadratic and cubic polynomials of elevation as predictor variables, and grey areas are 95% credible intervals for model predictions.

High atmospheric N deposition was related to low values of all six measures of landscape-scale plant diversity (table 2). Standardized atmospheric N deposition had the strongest negative relation to PD, and PD loss in relation to a natural N deposition without human influence (historic scenario 1) was estimated at 19% (median; 95% credible interval: 11–26%; figure 1*a*); based on historic scenario 2, PD loss was estimated at 11% as compared with the year 1880 (median; 95% credible interval: 6–15%; figure 1*b*). Atmospheric N deposition had the weakest negative relation to total species richness (median loss, scenario 1: 5%; 95% credible interval: 2–8%; median loss, scenario 2: 3%; 95% credible interval: 1–4%). Plant diversity was also predicted by other factors (table 2); in particular, community uniqueness increased with elevation and FD decreased with elevation, while the three measures of species diversity showed highest values at intermediate elevations (figure 2).

**Table 2.** Results of LMs on total species richness (Total SR), number of oligotrophic species (Oligo SR), number of target species for conservation in Swiss agriculture (Agri SR), community uniqueness, functional diversity (FD) and phylogentic diversity (PD) at 381 1 km^2^ study plots. Given are non-standardized parameter estimates for linear (L) and quadratic (Q) terms of the predictor variables listed in table 1 (‘<0’ for estimates between −0.001 and 0; ‘>0’ for estimates between 0 and 0.001).

predictor variable	Total SR	Oligo SR	Agri SR	uniqueness	FD	PD
atm. N deposition (L)	−0.024***	−0.039***	−0.051***	−0.004**	−0.037**	−271.973***
atm. N deposition (Q)	>0**	>0	>0**	>0**	0.001***	4.620***
elevation (L)	>0***	0.001***	0.001***	<0***	−0.001***	2.000***
elevation (Q)	<0***	<0***	<0***	>0***	<0	−0.002***
agricultural area (L)	0.005***	0.001	0.005**	−0.001***	0.013***	32.650***
agricultural area (Q)	<0***	<0***	<0***	>0***	<0***	−0.579***
open woodland area (L)	0.013***	0.012**	0.016***	−0.003***	0.007	101.405***
open woodland area (Q)	<0***	<0**	<0***	>0***	<0	−1.985***
calcareous substrate (L)	0.006***	0.006***	0.007***	0.000	0.005**	27.157**
calcareous substrate (Q)	<0**	<0*	<0	>0	<0**	−0.135
temperature var. (L)	0.004***	0.011***	0.004**	<0	0.009***	47.600***
temperature var. (Q)	<0***	<0***	<0**	>0	<0***	−0.174***
s.d. creek lengths (L)	0.003**	0.002	0.004*	<0	0.006**	31.422**
s.d. creek lengths (Q)	<0	<0	<0	>0	<0**	−0.256
total deviance explained (%)	73.2	80.0	72.1	86.5	89.2	74.6

*p<0.1, **p<0.05, ***p<0.01.

## 5. Discussion

Our six measures of plant diversity showed strong differences in elevational patterns, suggesting that they indeed reflected different aspects of plant diversity. We found unimodal distributions with mid-elevation peaks in total species richness, oligotrophic species richness, number of target species for conservation in Swiss agriculture and PD, while FD linearly decreased and species uniqueness increased with elevation. These results suggest that elevational patterns in biodiversity not only depend on geographical region, climate zone or spatial scale as was found in previous studies [39,40], but also depend on the particular measure of diversity. In spite of the diverse nature of the six measures, however, low values of plant diversity were consistently associated with high atmospheric N deposition.

To compare the size of the negative effects of N deposition among the measures of plant diversity, we compared current plant diversity in Swiss landscapes with predicted plant diversity for two historic N deposition scenarios. This approach was similar to studies using bioclimatic envelope models to predict the response of biodiversity to climate change based on different climate change scenarios [41]. In the first historic scenario, we assumed a homogeneous distribution of N deposition for landscapes without human influence. For the second historic scenario, we assumed spatially varying N deposition predicted for the year 1880, during industrialization in Europe [13]. We found that current atmospheric N deposition is 17 times the assumed natural N deposition without human influence, and about 2.5 times the assumed atmospheric N deposition in 1880. Even though the two scenarios of historic N deposition thus largely differed with respect to quantity and spatial distribution of N deposition, we found a current loss of plant diversity due to N deposition in comparison to both historic scenarios. Also the relative order in the size of the effects on different measures of plant diversity was the same for both historic scenarios. We are thus confident that our results are sufficiently robust with regard to our assumptions on historic N deposition. The results suggest that while atmospheric N deposition was already quite high during industrialization in Europe as compared with a natural N deposition, there still has been a considerable loss in plant diversity due to atmospheric N deposition in the last 130 years.

By comparing plant diversity within and between small-sized study plots, Chalcraft *et al.* [10] found that N enrichment generally reduced biodiversity at local (within a plot) and regional (within and among plots) scales, but effect sizes varied substantially among experiments. In our study, the plots were much larger, each potentially containing many plots of a size as investigated in Chalcraft *et al.* [10]. Therefore, our within-plot diversity can be considered as a direct measure of the regional-scale diversity as investigated by Chalcraft *et al.* [10]. By comparing the recorded species among our 381 large-scale study plots for calculating community uniqueness, we added a new spatial scale, the landscape scale. Even at this large spatial scale, increased atmospheric N deposition was related to low plant diversity. This is evidence that atmospheric N deposition has negative effects on plant diversity not only at smaller plots as was shown in many previous studies [1], including our earlier results from 122 10 m^2^ plots distributed over Swiss mountain hay meadows [31], but also when considering different measures of plant diversity at a regional scale ([10] and our study) and even at the landscape scale (our results on community uniqueness) covering many different habitat types over an elevational range of 2900 m.

Conservation measures usually take place at relatively large scales (10–1000 ha) in landscapes consisting of different habitat mosaics that are considerably larger than the scale of most experimental studies [10]. Understanding the effect of N deposition at spatial scales at which ecosystems are managed and sites of conservation value are selected is thus important to develop conservation strategies [7]. Our study revealed clear and consistent evidence of negative impacts of atmospheric N deposition on plant diversity across different landscapes such as the Swiss lowlands that are shaped by intense human activity or the more natural alpine landscapes at higher elevation.

As usual, observational results need to be interpreted with caution. However, manipulative experiments are hardly applicable at the large spatial scale covered in our study. Also, experimental studies often examined communities differing markedly from those in natural landscapes [7]. Large-scale observational studies are thus needed to complement experimental results from small-scale plots, to illustrate effects of N enrichment in entire landscapes that are of particular concern for global change biology and conservation [10].

We found the strongest negative relation between atmospheric N deposition and PD. Plant communities with high PD often perform better in terms of ecosystem stability and biomass production than communities with lower PD ([29,42], but see [43]). Thus, large amounts of deposited atmospheric N may reduce ecosystem services via decreasing PD [5]. Because ecosystem services are typically provided over larger areas [7] like those investigated in this study, we suggest that in a densely populated country like Switzerland, anthropogenic atmospheric N deposition is threatening plant diversity and thus ecosystem stability and functioning.

## Data accessibility

Data are openly published in the Dryad Digital Repository (doi:10.5061/dryad.nf0kk).
